# Ultrasonographic Assessment of Meniscus Damage in the Context of Clinical Manifestations

**DOI:** 10.3390/medicina61081339

**Published:** 2025-07-24

**Authors:** Tomasz Poboży, Wojciech Konarski, Kacper Janowski, Klaudia Michalak, Kamil Poboży, Julia Domańska-Poboża

**Affiliations:** 1 Independent Researcher, Sarmacka 16/32, 02-972 Warszawa, Poland; tomasz.pobozy@onet.pl; 2 Medical Rehabilitation Center, Sobieskiego 47D, 05-120 Legionowo, Poland; wkonarski@poczta.onet.pl; 3 Department of Internal Medicine, Specialist Regional Hospital, 06-400 Ciechanow, Poland; kld.michalak@gmail.com; 4 Department of Neurosurgery, Brodnowski Masovian Hospital, 03-242 Warsaw, Poland; pobozykamil@gmail.com; 5 Department of Rheumatology, National Institute of Geriatrics, Rheumatology and Rehabilitation, 02-637 Warsaw, Poland; julia-domanska03@wp.pl

**Keywords:** meniscus, meniscus tears, knee joint, meniscus pathologies, meniscus cysts, degenerative meniscus tears, ultrasound

## Abstract

*Background and Objectives*: Meniscal pathologies are common abnormalities of the knee joint and a frequent cause of knee pain. Prompt and accurate diagnosis is essential to ensure appropriate treatment. Ultrasonography is increasingly used due to its accessibility, cost- and time-efficiency, and capacity for dynamic assessment. This study aimed to evaluate the usefulness of ultrasonography in identifying specific types of meniscal tears and to assess their frequency of occurrence. *Materials and Methods*: A retrospective study was conducted to assess the frequency and sonographic appearance of various meniscal pathologies. The study population included all patients who underwent ultrasonographic examination of the knee in our clinic over one year for various indications (*n* = 430). Archived ultrasound images were retrospectively reviewed and analyzed. *Results*: Meniscal pathologies were identified in 134 patients. The findings included 95 cases of degenerative lesions (70.9%), 18 meniscal cyst-related pathologies (13.4%), 8 complex tears (6.0%), 5 flap tears (3.7%), 3 vertical pericapsular tears (2.2%), 3 partial thickness tears (2.2%), and 2 bucket-handle-type tears (1.5%). Each lesion type was characterized and illustrated through representative ultrasound images. *Conclusions*: Ultrasound imaging of meniscal pathology offers a valuable diagnostic option. By characterizing and visually documenting different meniscal lesions, this study highlights the practical potential of ultrasonography in routine clinical settings. These findings may enhance diagnostic accuracy and guide more targeted management strategies. Moreover, the results contribute to the expanding body of research on musculoskeletal ultrasonography and may encourage broader adoption of ultrasound in orthopedic diagnostics.

## 1. Introduction

### 1.1. Background

The pathologies of the menisci are among the most common pathologies of the knee joint and are a common reason for patients reporting pain. Ultrasound examination (US) is a widely available, non-invasive, relatively cost- and time-efficient examination that also enables functional evaluation of the knee joint, including meniscal pathologies. The aim of this study is to characterize the ultrasound image of individual types of meniscal pathologies, illustrate them with images from the authors’ clinical practice, and to assess the frequency of individual types of the pathology with retrospective analysis of images from ultrasound examinations.

### 1.2. The Most Important Practical Aspects of Meniscus Anatomy

There are several methods used to describe meniscal anatomy. Based on the meniscus width, the first method divides it into three zones: the outer (red) zone, the intermediate, and the free margin zone. From a practical point of view, we recommend dividing the meniscus into three parts: the anterior, middle, and posterior.

Menisci are connected to the joint capsule along most of their length. The horns of the menisci are connected directly to the tibia. The menisci are closely connected to the tibia through short meniscotibial ligaments, which constitute a direct reinforcement of the joint capsule. The middle part of the length of the medial meniscus is slightly more strongly connected to the femur by the meniscofemoral ligament, which constitutes a deep layer of the medial collateral ligament (MCL). When assessing the lateral meniscus, it should be borne in mind that on the border of the middle 1/3 and posterior 1/3 (the outer zone), there is a hiatus of the tendon of the popliteal muscle [1,2,3,4,5,6].

### 1.3. The Method of Examination and the Physiological Image of the Meniscus in Ultrasonography

As a standard during US examination, we evaluate the meniscus by holding a linear probe at the height of the joint line perpendicularly to the joint gap. We propose starting the assessment at the middle part of the meniscus and moving the probe along the joint space forward and backward, assessing the middle, anterior, and posterior parts, respectively. We assess the middle and anterior part of the meniscus with the limb bent to a 90 degree angle while the patient is lying in the supine position. The posterior part of the meniscus is assessed when the patient lies in the prone position. The authors prefer the terms the posterior and anterior parts, not the posterior and anterior horns of the meniscus, because we believe it is only possible to properly visualize and evaluate only a limited part of the meniscus that adheres to the joint capsule.

The horns themselves are situated away from the capsule, attached directly to the tibia, so the assessment of the horns may not be objective, particularly regarding the anterior horns. In the area of the anterior horns, the space between the meniscus and the joint capsule is filled with a Hoffa fat pad, with echogenicity similar to the echogenicity of the meniscus. Hence, it is difficult to accurately assess the location of the border between the meniscus and the fat pad in the US. This border, characterized by a heterogeneous echogenicity due to the proximity of the anterior meniscal horn and the fat pad, might be erroneously described as damage to the meniscus. In clinical practice, the authors face an excessive number of false-positive US diagnoses of anterior meniscus lesions. In contrast, the authors rarely observe damage to the anterior horn of the meniscus during arthroscopy.

When assessing the posterior part of the lateral meniscus, it should be remembered that it is located a little deeper in the joint.

The meniscus assessment in a plane parallel to the joint gap is more complicated. Most clinicians routinely do not perform it, but it may be valuable in certain situations.

A meniscus tear can sometimes be observed only during functional examination (flexion-extension, rotational movements).

### 1.4. Diagnostic Methods in Meniscal Pathologies Imaging

Ultrasonography is regarded as a valuable tool used to identify meniscal pathologies. Although, compared to MRI, it offers greater availability, lower examination costs, and the ability to assess during movement, the diagnostic potential of ultrasound may still not be fully utilized. The effectiveness of diagnostic ultrasound in identifying meniscal injuries, based on separate studies, shows comparable or superior diagnostic abilities compared to MRI when each is evaluated against arthroscopy [7,8]. Recent research suggests that migrating to the US as a primary tool is suitable for assessing certain meniscal injuries when used by skilled operators. Acknowledging its constraints, such as limited visibility in specific regions and issues like ultrasound artifacts, is crucial for the careful and accurate use of ultrasound in detecting meniscal tears [9]. A study conducted by Ahmadi et al. [10] demonstrated that point of care ultrasound (POCUS) could be an accurate and reliable diagnostic tool as an alternative to MRI in detecting medial meniscal tears. The authors concluded it might be effective and could conduct immediate investigation to guide further modalities in patients with acute knee trauma. In the study (157 patients included) 56.7% of medial meniscal tears were detected using arthroscopy as the preferred method. MRI diagnostic accuracy was 93%. POCUS accuracy for detecting medial meniscal tears was 89.2%.

## 2. Materials and Methods

The PubMed database was used to gather data regarding menisci pathologies. The collected papers have been reviewed and summarized in order to characterize meniscus pathologies. The high-quality ultrasound images from the authors’ clinical practice were obtained and used for the better visualization of the topics discussed.

A retrospective study with access to medical records has been conducted to assess the frequencies of the common types of meniscus pathologies in the clinical practice. The study population consisted of all people who underwent ultrasound examination of the knee joint at the clinic during one year for various reasons. The group of participants comprised 66.5% females and 33.5% males, ranging in age from 16 to 82 years. The participants were referred for diagnostic evaluation due to pain-related symptoms (70%) or a history of trauma (30%).

The inclusion criteria involved the patient’s signing of consent during the study. The exclusion criterion was lack of consent; however, all patients we examined gave their consent for the use of images. The linear 3–12 MHz probe was used in the study. A total of 430 patients has been included. In every case, the US images were retrospectively analyzed by an experienced orthopedist to select patients with meniscus pathologies. The number of 134 patients was selected this way. The images were further divided according to the type of the pathology.

Patients in whom the meniscal cysts had been identified constituted a separate group, regardless of the cooccurring meniscal pathologies.

## 3. Results

### 3.1. Most Common Pathologies of Menisci

#### 3.1.1. Meniscus Tears

It is widely known that meniscus damage can take various forms, from intrasubstance tear and free-margin delamination, through radial, vertical, horizontal flap formation, or parrot beak tears, to bucket-handle-type damage, which, when displaced, can cause a joint block. When reviewing the patient’s medical history, the presence of trauma, the circumstances of the first symptoms’ onset, their duration, association with physical activity, and its type should be considered in particular. During the physical examination, local soreness over the joint gap or positive meniscal tests might be present (McMurray, Apley, Thessaly, duck waddle, among others). Injury may be accompanied by other symptoms such as a joint block. It is necessary to be aware of less typical symptoms such as swelling of the joint and possible coexisting damage of other knee structures, which might also result from the trauma [11,12,13,14,15,16,17].

**Figure 1:**
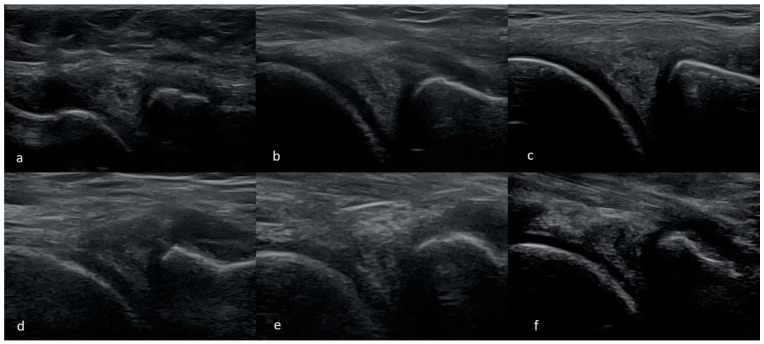
Images of intrasubsantional damages of menisci (**a**) corresponding to type I lesion seen in MRI; (**b**,**c**) to type II; (**d**–**f**) to type III.

The most commonly seen meniscus tears are intrasubstance, horizontal, or oblique tears. They are found mainly in the posterior 1/3 or between the posterior and the middle 1/3 of the medial meniscus. Less frequently, the tear is observed in the middle or posterior 1/3 of the lateral meniscus. It can present as mid-substance damage only where the delamination does not contact the inside of the joint, which corresponds to type I and II tears in the MRI. More commonly, however, the injury compromises a larger part of the width of the meniscus. In such cases, the tear connects to the inside of the joint, usually the inferior surface of the meniscus. That corresponds to the image of type III damage in MRI. In our opinion, this type of damage is the easiest to recognize on the US examination. This type of damage is usually seen in people over 40 years, typically results from age-related degenerative processes, and is usually not associated with one specific injury. Although this type of damage might cause pain in the medial part of the knee joint, it is often solely an incidental finding that does not cause clinical symptoms (Figure 1).

**Figure 2:**
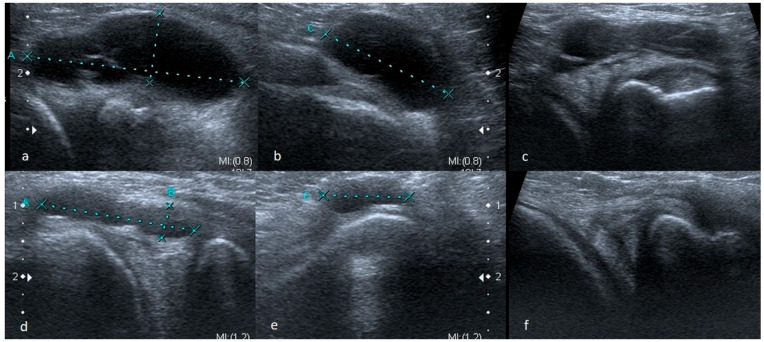
Two series of scans (I (**a**–**c**), II (**d**–**f**)) showing large meniscal cysts; in each case horizontal tear was observed (we believe that whenever a meniscus cyst is present, there is a coexisting horizontal or oblique tear in meniscus, and that the cyst forms as a hernia of the articular capsule, which is the result of increased pressure of the synovial fluid pressing against the capsule through the fissure of tear).

In some cases, meniscus tears are revealed only during a functional examination. Horizontal or oblique tears, more often than other meniscus damage, may be accompanied by the presence of meniscal cysts (Figure 2).

#### 3.1.2. Meniscus Cysts

**Figure 3:**
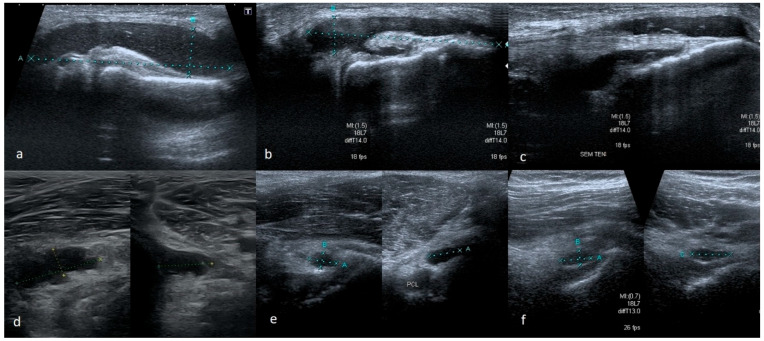
(**a**–**c**) A study showing large meniscal cyst descending along the semitendinosus tendon from the level of the joint to the attachment of the tendon to the pes anserius; (**d**–**f**) ganglions posterior to the PCL localized near posterolateral margin of the medial condyle of the femur—potentially posterior horn meniscal cyst.

Cysts can be of different sizes and locations. Sometimes they are situated only in the outer zone of the meniscus; sometimes, they are large and can descend peripherally, e.g., along the tendon of the semitendinosus muscle giving an image of a cyst (ganglion) of tendons forming pes anserinus. In our opinion, some of the changes described as ganglions in the region of the posterior cruciate ligament (PCL) originate from an injury to the posterior horn of the medial meniscus, especially if they adhere to the posterolateral margin of the medial condyle of the femur (Figure 3).

If a cyst accompanies a meniscus tear, the patient usually experiences pain. It appears most often during physical activity. In the physical examination, the leading symptom is tenderness. In the presence of cysts, meniscal tests are not always positive [18,19,20,21,22,23].

**Figure 4:**
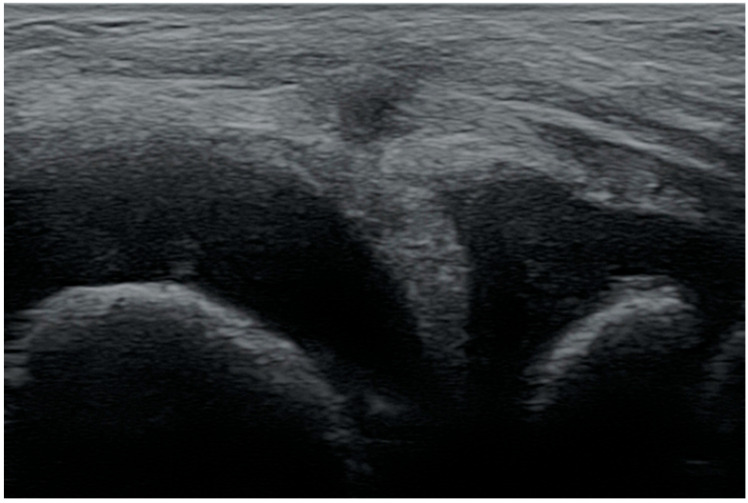
A scan of the posteromedial part of the healthy medial meniscus of a girl aged 5.

When assessing the posterior part of the meniscus in the pediatric population, it should be remembered that healthy tissue may resemble an intrasubstance tear. That is due to the greater number of nerve endings, the presence of blood vessels in the outer zone, and lower density of connective tissue in children [24,25] (Figure 4).

**Figure 5:**
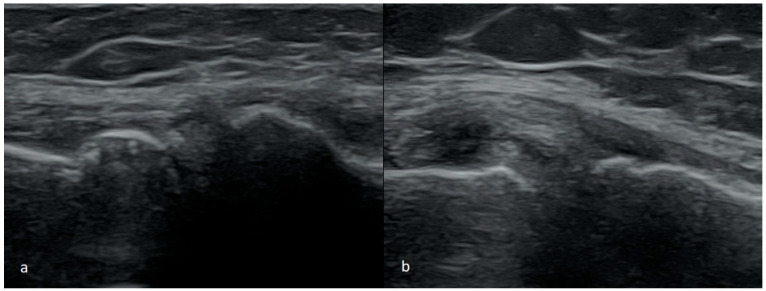
(**a**) Partial, (**b**) full thickness peripheral vertical meniscal tear.

The ease of recognizing a vertical pericapsular tear depends on the size of the injury and the degree of mobility or displacement of the damaged part of the meniscus. Larger, unstable damage is theoretically easier to assess, and stable damage is usually not accompanied by clinical signs. Nonetheless, small stable tears (less than 1 cm in length) may remain invisible on the US or only be observed during a functional examination. In contrast, if the meniscus tissue is torn over a longer distance, we can observe the gap between the meniscus and the capsule (Figure 5).

#### 3.1.3. Bucket-Handle Tears

US imaging of the bucket-handle tear, where the damaged fragment is displaced towards the intercondylar fossa, causes the most problems and often remains unrecognized. The difficulty arises from an increased amount of dense synovial fluid or hematoma (especially in recent injuries), which commonly accompanies damage of this kind. If a dense fluid or hematoma is present in the joint, it is often characterized by higher echogenicity. Observing such fluid near the meniscus tear, the examiner may think he is observing an intact meniscus. In order to avoid misjudgment in such a situation, we suggest assessing the meniscus by slowly moving the probe along the entire joint. It should be moved from the anterior part to the middle and then from the posterior to the middle part. Through the exam, it should be kept along the long axis of the limb.

**Figure 6:**
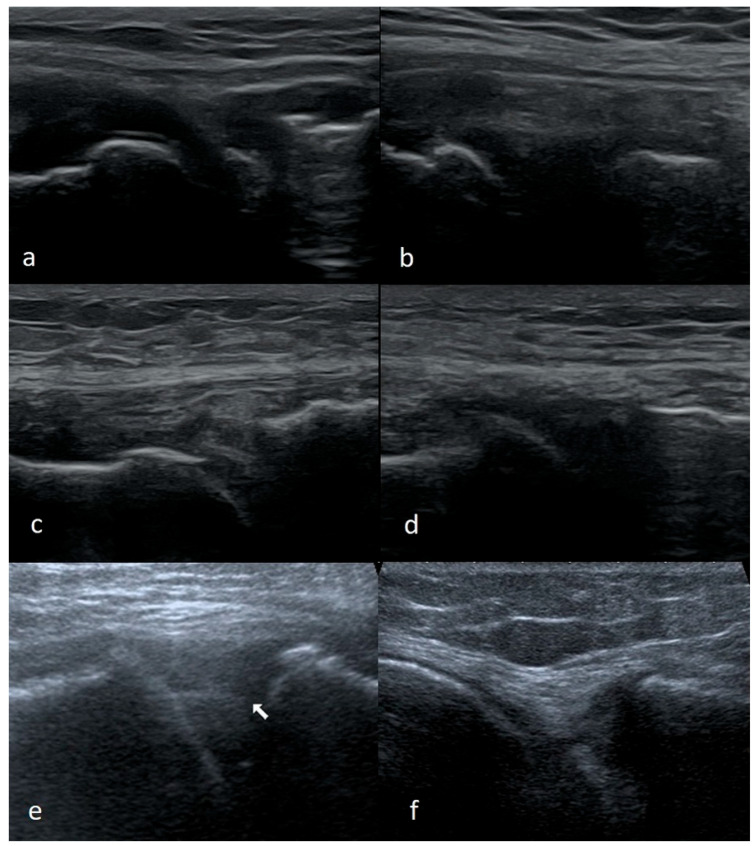
Two cases of bucket-handle tear (**a**–**d**). (**a**,**c**) Scans showing abnormal echostructure of meniscus where the body of meniscus is twisted, (**b**,**d**) the absence of the body of the meniscus at the joint capsule in the place where it is displaced towards the inside of the joint; (**e**,**f**) scans showing damaged menisci (two cases) detached from the joint capsule, but not completely displaced into the intercondylar fossa; both are visible at a short distance from the capsule.

Some portion of the meniscus situated anteriorly and posteriorly remains intact in these type of tears. When the damaged portion is displaced into the intercondylar fossa, the intermediate part (between displaced and non-displaced fragments) of the damaged meniscus is twisted. When such injury is present, during the US examination, moving the probe from the area close to one of the horns, the undamaged part of the meniscus is first observed. Moving the probe further, the echostructure of the meniscus becomes altered because the meniscus is twisted at the point of eversion. Sometimes we can see within a short distance the meniscus sticking out of the joint capsule, and then the zone where the meniscus does not adhere to the capsule; we again observe the area of disturbed echostructure at the site of connection of the other end of the damage zone with the rest of the meniscus and finally we reach the other side of the intact part of the meniscus. The zone where the meniscus does not adhere to the capsule can be filled with low-echogenicity fluid, which makes the diagnosis simpler, or the fluid can be given a higher echogenicity, which may imitate the presence of the meniscus (Figure 6).

**Figure 7:**
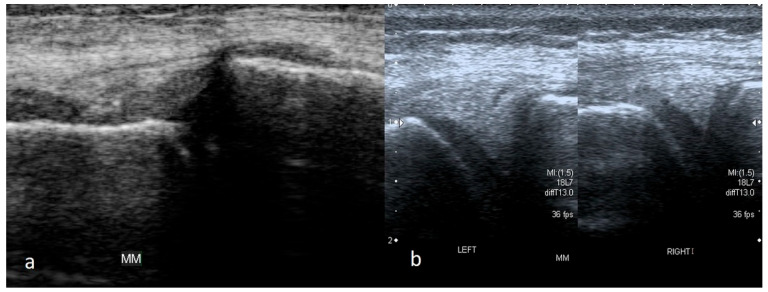
(**a**) A remaining-along-joint-capsule part of meniscus takes trapezoid shape; (**b**) the comparative study showing on the right side a much larger meniscus with a small intrasubstance tear; on the left side the meniscus has its proper triangular shape on the cross-section, but it is much smaller in size—such an image indicates its chronic damage with the loss of its body or probably with the displacement of its fragment into the intercondylar notch.

Depending on the part of the width of the meniscus where the damage occurs, more or less of the meniscus substance may remain attached to the joint capsule. In a recent injury, a part of the meniscus attached to the joint capsule would take a trapezoid shape instead of a triangle on the cross-section scans. In chronic damage, there is usually no increased fluid in the joint. The remaining part of the meniscus in the outer zone may be deformed, and its shape may change from trapezoidal to triangular. It is essential to compare the meniscus with the meniscus in the contralateral knee in such a situation. The size of the meniscus on the affected side will be significantly smaller (Figure 7).

**Figure 8:**
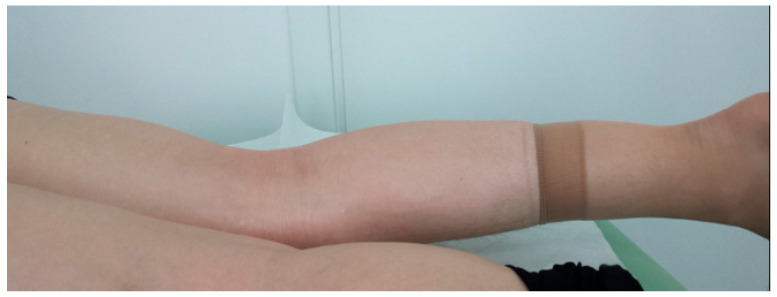
“True” joint block does not disappear in the prone position.

Bucket-handle tears have some characteristic clinical manifestations. They usually arise after injury—usually, after sudden twists during physical activity, e.g., football in summer, skiing in winter, but sometimes due to trivial injuries such as rising from a squatting position when the limb is twisted. In addition to pain in the meniscus projection, a typical symptom is a joint block. The block is a common characteristic of meniscus damage and is characterized by a lack of extension beyond 5–10 degrees; this deficit does not decrease when the patient lies in the prone position (Figure 8).

Undoubtedly, other causes of a joint block have to be considered in a differential diagnosis. A greater limitation of extension (of about 30 degrees) with significant limitation of flexion (so-called “pseudoblock”) rather indicates damage to other structures or several structures at the same time (e.g., cruciate ligaments). In contrast, bucket-handle tears, besides a joint block, are also characterized by positive meniscal tests [11,26,27].

**Figure 9:**
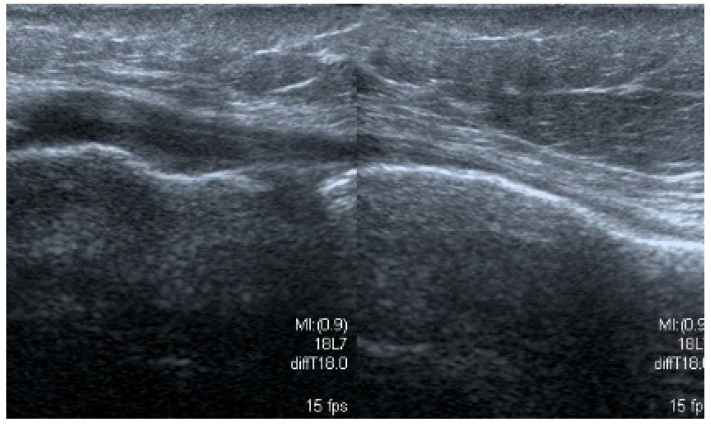
A longitudinal scan of the medial collateral ligament, hypoechoic scar formation in the proximal part of the ligament.

At this point, it is worth discussing the differential diagnosis of the meniscus and medial collateral ligament (MCL) injury. When dealing with the consequence of trauma to the medial side of the joint, distinguishing between these two can be particularly problematic. Meniscal tests may be positive in an acute injury setting with both of the above lesions. When the injury involves the MCL, it usually affects its femoral attachment—hence the pain is most often located above the joint line at the level of the medial epicondyle of the femur. In contrast, in meniscus injury, the pain is localized at the height of the joint line. In the case of damage to the MCL, especially partial damage, the pain intensifies when trying to valgus the joint (it does not occur with the meniscal damage). The pain may be less severe following complete MCL rupture, but we observe the medial instability instead. It is important to remember that instability in the frontal plane should be examined in slight flexion of the knee joint (up to about 30 degrees). Fortunately, in the US examination, identification of damage to the MCL injuries is simple (Figure 9).

#### 3.1.4. Meniscal Flap Tears

Quite often, we observe the flap tears. On the US, when the damaged part moves towards the inside of the joint, meniscus tissue remaining in the outer zone might be reduced in size. Clinically, the patient usually presents with the symptoms of periodic blockage of the joint, accompanied by periodically intensifying pain. Meniscal tests may be positive; less often, we observe a deficit of extension.

**Figure 10:**
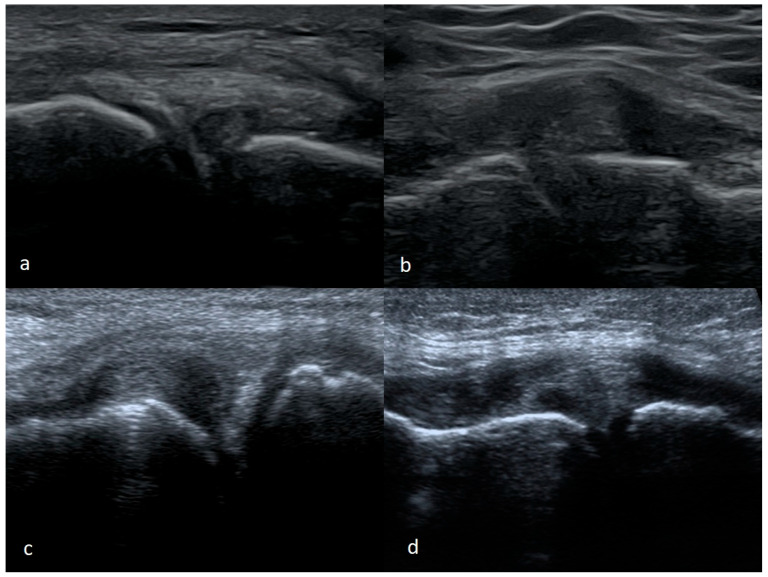
Flap tear (**a**,**b**) scans showing the flap turned under the surface of the meniscus, (**c**,**d**) flap turned up above the surface of meniscus.

A specific situation occurs when the flap tear is wrapped under the surface of the meniscus or above it (Figure 10).

The authors observed this type of injury only with the medial meniscus. In the first situation, the torn fragment is often wrapped to become locked between the lower surface of the meniscus, the articular capsule, and the medial surface of the upper part of the medial tibial condyle. In the second case, clinical symptoms are similar to the bucket-handle tear. The most typical symptom is pain, intensifying during physical activity, and, in the physical examination, there is a pronounced tenderness on deep palpation at the joint-line level.

#### 3.1.5. Degenerative Changes of Menisci

**Figure 11:**
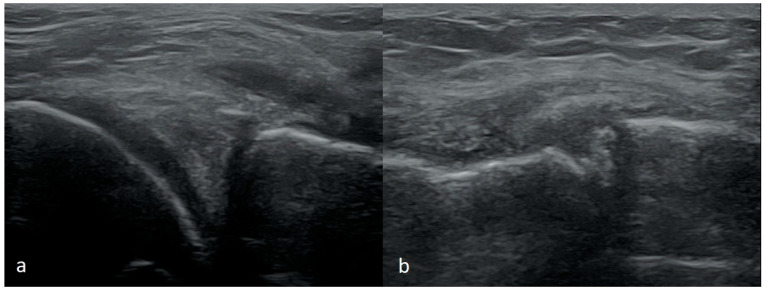
(**a**) A simple degeneration of the meniscus; (**b**) a degeneration of the meniscus with the presence of calcifications inside its body.

The degenerative changes of the meniscus are characterized by heterogeneous echogenicity on US. In case they present without visible fissures of the tear, they should not be perceived as the cause of pain (Figure 11).

**Figure 12:**
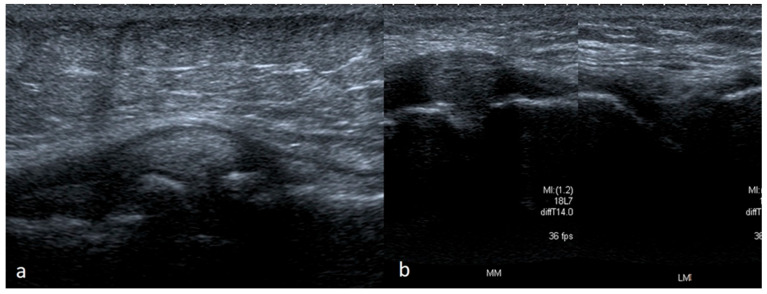
Meniscal extrusion. (**a**) Posteromedial part of medial meniscus; (**b**) on the left—significant extrusion of the medial meniscus with modeling of the medial collateral ligament, on the right—an image of the healthy lateral meniscus.

At an advanced stage of meniscus degeneration, fibrosis of the free margin appears, which is unlikely to be assessed objectively in the US. With more advanced degeneration, we see the tears mentioned above and the features of extrusion (displacement beyond the joint space), which typically affect the middle parts of the meniscus. Extrusion of the medial meniscus can directly cause pain if the meniscus tightens and irritates MCL. The lateral meniscus does not tighten the lateral longitudinal ligament, even with significant extrusion, because it is distant from the joint capsule. In contrast, MCL constitutes a direct reinforcement of the articular capsule on the medial side. Meniscus extrusion often accompanies degenerative changes, which is why there is often a conflict with the protruding meniscus and MCL and the osteophytes at the margins of the joint space. Degenerative tears are often diagnosed in older patients (usually over 40 years of age) [28,29,30,31,32,33,34,35] (Figure 12).

The assessment of the meniscus after its repair is a separate issue and goes beyond the framework of this paper.

### 3.2. The Results of the Retrospective Analysis

Out of the 430 patients included in the study, 134 have been identified as patients with meniscus pathologies. Of these patients, the degenerative lesions were identified in 95 cases (70.9%). A total of 18 cases was classified as the cystic lesions (13.4%). Complex tears occurred in eight patients (6.0%). In five patients flap tears were identified (3.7%), in three patients—a vertical pericapsular tear (2.2%), in three patients—a partial (partial thickness) tear (2.2%), and in two patients—a bucket-handle-type tear (1.5%) (Table 1). Patients with meniscus cysts were classified as a separate group, regardless of cooccurring pathologies (mostly horizontal or complex tears).

## 4. Discussion

Despite the growing availability of MRI, there is still a place for US in the assessment of orthopedic injuries. US ensures capability to evaluate and examine during motion, reduced costs, and may still not be utilized to its full potential. Recent studies have shown US high effectiveness in meniscal pathologies examination (compared to MRI or arthroscopy) and have suggested using US as a primary tool in certain cases [7,8]. To achieve satisfactory results, it is important to remember that US should be conducted and interpreted by individuals with appropriate expertise and knowledge due to the occurring artifacts and limitations. Though it is not always possible to establish a final diagnosis, it might guide further the diagnostic process or treatment progress. The use of ultrasonography at an early stage of diagnosis can help determine appropriate treatment and provide better guidance for further non-invasive or invasive interventions. Recent studies have shown that ultrasonography still remains an underutilized diagnostic tool [36,37]. Although magnetic resonance imaging is an accurate and commonly chosen method for diagnosing meniscal pathologies, it is important to remember its limitations. Ultrasonography has been suggested as an effective alternative, especially following meniscal injuries. Ultrasonography serves as an effective screening method for the early identification of meniscal injuries in comparison to MRI, and it might even provide benefits over MRI, especially in situations where MRI cannot be used or is not recommended. The use of modern, portable ultrasound devices additionally allows this diagnostic method to be applied at the site of injury [8]. We express hope that our article will systematize the knowledge about the US imaging of meniscus injuries. It may recall or bring closer the clinical signs to diagnosticians, and some orthopedists may improve their diagnostic skills [38,39,40,41,42,43,44,45,46,47,48].

## Figures and Tables

**Table 1:** Types and characteristics of meniscal pathologies.

Pathology	% of Identified Lesions	Echogenicity Pattern	Mobility/Pain
Degenerative lesions	70.9	- Heterogeneous echogenicity - Meniscal extrusion (displacement beyond the joint space)	- Extrusion of the medial meniscus can cause pain
Cystic lesions	13.4	- Well-defined, anechoic areas with thin, smooth walls	- Patient usually experiences pain if a cyst accompanies a meniscus tear - Tenderness - Meniscal tests are not always positive
Complex tears	6.0	- May vary depending on the type of lesion	- May vary depending on the type of lesion
Flap tears	3.7	- The damaged part moves towards the inside of the joint - Meniscus tissue remaining in the outer zone might be reduced in size	- Periodic blockage of the joint - Positive meniscal tests - Deficit of extension
Vertical pericapsular tears	2.2	- The gap between the meniscus and the capsule might be observed	- Stable damage is usually not accompanied by clinical signs
Partial thickness tears	2.2	- Intrasubstance, horizontal or oblique tears - Found mainly in the posterior 1/3 or between the posterior and the middle 1/3 of the medial meniscus	- Pain in the medial part of the knee joint - Often incidental finding that does not cause clinical symptoms
Bucket-handle-type tears	1.5	- Twisted body of the meniscus - Absence of the body of the meniscus at the joint capsule in the place where it is displaced towards the inside of the joint - Deformation of part of the meniscus in the outer zone (its shape may change from trapezoidal to triangular)	- Pain in the meniscus projection, - Joint block characterized by a lack of extension beyond 5–10 degrees (does not decrease in the prone position) - Positive meniscal tests

## Data Availability

The original contributions presented in this study are included in the article. Further inquiries can be directed to the corresponding author(s).
